# Activity dependent modulation of synaptic transmission by presynaptic calcium stores: A dichotomy of short-term depression and facilitation

**DOI:** 10.1186/1471-2202-14-S1-P351

**Published:** 2013-07-08

**Authors:** Suhita Nadkarni, Thomas Bartol, Herbert Levine, Terrence Sejnowski

**Affiliations:** 1 Wellcome Trust-DBT Intermediate Fellow Indian Institute of Science Education and Research, Pune, India; 2 Salk Institute for Biological Studies, La Jolla, 92037, USA; 3 Rice University, Houston Texas, 77030, USA

## 

Ongoing electrical activity can initiate a positive feedback loop via Inositol Triphosphate Receptors (IP_3_Rs) and Ryanodine Receptors (RyRs) on the Endoplasmic Receptors (ER) that can lead to release of calcium from the ER. We investigated how the presence of this additional source of calcium in the presynaptic terminals in addition to the Voltage Dependent Calcium Channels (VDCCs) can regulate synaptic transmission. We carried out 3D Monte Carlo simulations of all the molecular interactions that govern transmitter release in a 1) Canonical CA3-CA1 synapse 2) A synapse reconstructed from serial section Transmission Electron Microscope images. The relatively simple geometry of CA3-CA1 synapses allows activity-dependent local calcium at the active zone and the related transmitter release profiles to be quantitatively analyzed. In paired-pulse stimulation, the presence of the molecular pathways that regulate the calcium stores increased the calcium buffering capacity of the synapse, which decreased release probability and enhanced paired-pulse facilitation. In contrast, a high-frequency stimulus could trigger the activation of presynaptic RyRs and Metabotropic Glutamate Receptors (mGluRs) leading to IP_3 _production and ultimately to release of calcium from the SER. IP_3_Rs and RyRs operated at a much slower time scale, on the order of seconds compared to the millisecond timescale of the VDCCs. This led to an increase in the basal level of intracellular calcium and enhanced transmitter release rates. In reconstructions of hippocampal neuropil, the ER appears in a majority of the presynaptic terminals in varying degrees of abundance. We further explored the functional implications of the range of ER geometries and therefore the calcium carrying capacity, observed in the synaptic ultrastructure and the effect of different arrangements between IP_3_Rs and VDCCs on synaptic plasticity. The synaptic ultrastructure precisely orchestrated the degree of facilitation and depression at the synapse and the existence of presynaptic calcium stores provided the synapse with an additional intrinsic time scale that could be regulated by activity.

